# IgG, IgM, and Nonstructural Protein 1 Response Profiles after Receipt of Tetravalent Dengue Vaccine TAK-003 in a Phase 2 Randomized Controlled Trial

**DOI:** 10.4269/ajtmh.23-0549

**Published:** 2024-05-28

**Authors:** Jenny G. Low, Helen M. Oh, Yee-Sin Leo, Shirin Kalimuddin, Limin Wijaya, Junxiong Pang, Tau Hong Lee, Kelley J. Moss, Manja Brose, Vianney Tricou

**Affiliations:** ^1^ Department of Infectious Diseases, Singapore General Hospital, Singapore;; ^2^ Program in Emerging Infectious Diseases, Duke-NUS Medical School, Singapore;; ^3^ Changi General Hospital, Singapore;; ^4^ National Centre for Infectious Diseases, Singapore;; ^5^ Saw Swee Hock School of Public Health, National University of Singapore, Singapore;; ^6^ SingHealth Duke-NUS Global Health Institute, Duke-NUS Medical School, Singapore;; ^7^ Tan Tock Seng Hospital, Singapore;; ^8^ Takeda Development Center Americas, Inc., Cambridge, Massachusetts;; ^9^ Takeda Pharmaceuticals International AG, Zurich, Switzerland

## Abstract

The profiles of vaccine-induced dengue antibodies may differ from those produced following natural infection and could potentially interfere with the interpretation of diagnostic tests. We assessed anti-dengue IgG and IgM antibodies, and nonstructural protein 1 antigen profiles in the serum of adults who received a single dose of the tetravalent dengue vaccine TAK-003 as either an initially developed high-dose formulation or the standard approved formulation in a phase 2 study in Singapore (#NCT02425098). Immunoglobulin G and IgM profiles during the first 30 days postvaccination varied by baseline serostatus (microneutralization assay). Nonstructural protein 1 antigen was not detected in the serum of any participants. Vaccine-induced IgG and IgM antibodies can affect serological confirmation of subsequent dengue infection in vaccinees. These results highlight the limitations of using serological tests for dengue diagnosis, particularly in a postvaccination setting, and emphasize the need for more sensitive antigen- and molecular-based testing for accurate dengue diagnosis.

Dengue is a viral disease spread predominantly by the *Aedes aegypti* mosquito.^1^ Dengue incidence has increased substantially in recent decades and it is now considered a global threat to health.^1^^,^^2^ Dengue infection can lead to asymptomatic or mild illnesses, through to severe dengue, which can be fatal.^1^ As of June, more than 4 million cases of dengue and ∼4,000 dengue-related deaths were reported worldwide in 2023.^3^

As with most other viruses, including flaviviruses, the antibody response following dengue infection differs based on previous virus exposure. In dengue-naive individuals, dengue IgM is detectable in most patients after the first week of infection, circulating for ∼2 months.^4^^,^^5^ Low levels of dengue IgG are detectable from the second week postinfection, with lifelong persistence.^4^^,^^5^ In secondary dengue infections, the IgG response predominates, with titers increasing rapidly within the first few days of infection, whereas IgM levels remain low and are undetectable in many patients.^4^

Several methods are available for dengue diagnosis, including dengue-specific reverse transcription–polymerase chain reaction, nonstructural protein 1 (NS1)–based antigen testing, and detection of dengue-specific IgG and IgM using ELISA. Although serological ELISA tests are cheap and simple to perform, they are limited by several factors, including low antibody titers in the first days of infection, preexisting antibodies from previous infections, potential cross-reactivity induced by infection with other flaviviruses, and low or undetectable IgM levels in some patients with secondary dengue infections.^5^ In addition, vaccine-induced antibodies may confound IgG and IgM antibody test results, leading to false positives, as observed in vaccinees receiving the chimeric yellow fever dengue-tetravalent dengue vaccine (CYD-TDV).^6^^,^^7^ Detection of NS1, a glycoprotein secreted by infected cells during the acute phase, is also widely used for confirmation of dengue infection with high specificity. However, NS1 test sensitivity in nonprimary infections may be affected by virus–IgG complexes or the persistence of NS1-specific antibodies,^8^^,^^9^ which are produced as part of the anti-dengue immune response.^10^

TAK-003 is a tetravalent dengue vaccine based on a live-attenuated dengue virus 2 (DENV-2) virus (TDV-2) that provides the genetic backbone for the virus strains representing the three other serotypes, which were developed by replacing the TDV-2 pre-membrane and envelope genes with those from the wild-type DENV-1, DENV-3, and DENV-4 strains.^11^ The main attenuation markers of TDV-2, including an NS1 coding gene, are present in all four vaccine components.^11^^,^^12^ TAK-003 has been shown to be well tolerated and immunogenic.^13^ Furthermore, in a large-scale phase 3 study,^14^ vaccine efficacy from first dose to ∼4.5 years after the second dose was 61.2% against symptomatic dengue and 84.1% against dengue leading to hospitalization. To assess whether TAK-003–induced antibody and antigen profiles differ from those induced after natural infection and, therefore, may interfere with dengue diagnostics, the IgG and IgM antibody and NS1 antigen profiles were assessed in adults age 21 to 45 years living in Singapore (a dengue-endemic country) in a phase 2, double-blind, randomized study (#NCT02425098).^15^

Full details of the study design, eligibility criteria, and interventions have been published previously.^15^ In brief, eligible participants were randomized 1:1 to receive one dose of either the approved formulation of TAK-003 (referred to as TDV) or a high-dose formulation with 10-fold higher DENV-2 potency (HD-TDV) on day 1. Randomization was stratified by IgG status at screening (based on results of a locally performed dengue IgG indirect ELISA) to balance the number of participants who were dengue naive or dengue exposed. Immunogenicity was assessed against each serotype using a centrally performed microneutralization assay, with titers corresponding to a 50% decrease in plaque reduction (50% microneutralization assay [MNT_50_]). Participants were considered baseline seropositive if they had a reciprocal MNT_50_ titer ≥10 for one or more dengue serotypes. Blood samples were taken at baseline (day 1, prevaccination) and on days 15 and 30 for IgG; days 1, 7, 15, and 30 for IgM; and days 1, 5, 7, 9, 11, 15, 17, 21, and 30 for NS1. Testing was performed centrally using ELISAs (NS1 Antigen [NR-R10004] sandwich ELISA kit, Novatein Biosciences, Woburn, MA; Anti-dengue virus IgG [ab108728] and IgM [ab108729] capture ELISA kits, Abcam, Bristol, United Kingdom). The sensitivity and specificity of the IgG and IgM ELISAs in the context of acute dengue diagnosis have been reported previously.^16^ Results of our analysis are presented descriptively as the proportion of participants who tested positive per each ELISA and the associated two-sided 95% CIs, calculated using the Clopper-Pearson method. Positivity as identified with ELISAs was defined as the absorbance ratio (calculated as sample absorbance divided by manufacturer’s cutoff control absorbance) >1.1, per the manufacturer’s instructions. Mean absorbance ratios and associated 95% CIs were calculated for IgG and IgM by vaccine group and baseline MNT_50_ serostatus for each of the test days. Of note, these assays were not validated as quantitative ELISAs. Most analyses were performed using SAS v. 9.4 (SAS Institute, Cary, NC), with GraphPad Prism v. 9.4.1 (GraphPad Software, La Jolla, CA) used to calculate median ELISA absorbance ratios and associated 95% CIs.

Overall, 176 participants received HD-TDV and 175 received TDV. Baseline demographics were balanced across the two vaccine groups and were similar, regardless of baseline MNT_50_ serostatus, in each vaccine group.^15^

No participants tested positive for the NS1 antigen at any time point. The proportion of participants in each group with detectable IgG and IgM varied, but increased overall from baseline to day 30 (Figures 1 and 2), consistent with previously reported neutralizing antibody response profiles.^15^

**Figure 1.**
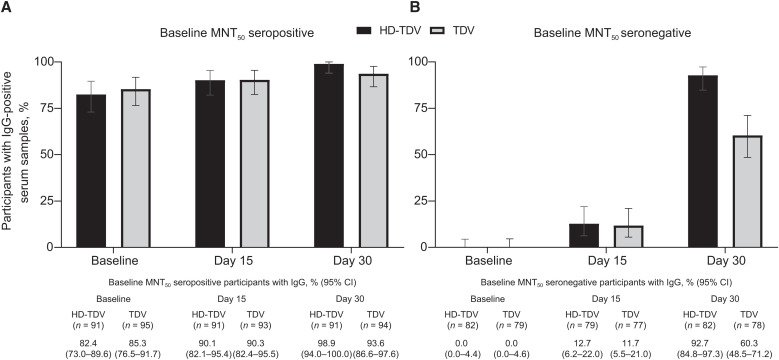
Proportion of participants with IgG-positive serum in (**A**) baseline 50% microneutralization assay (MNT_50_)–seropositive and (**B**) baseline MNT_50_–seronegative participants by vaccine dose group at baseline and days 15 and 30 (per-protocol set). The percentage of IgG-positive participants and 95% CI are shown. HD-TDV = high-dose formulation; TDV = approved formulation.

**Figure 2.**
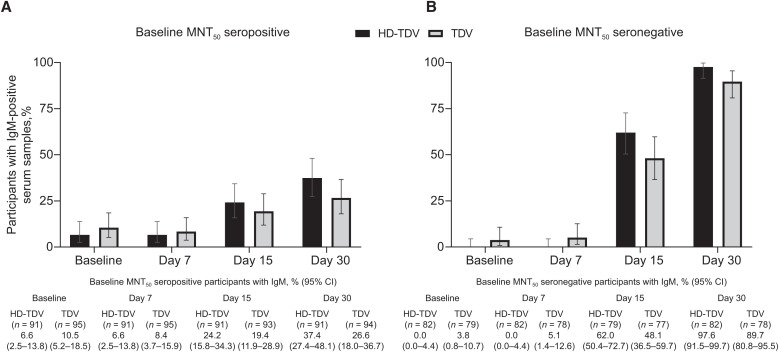
Proportion of participants with IgM-positive serum in (**A**) baseline 50% microneutralization assay (MNT_50_)–seropositive and (**B**) baseline MNT_50_–seronegative participants by vaccine dose group at baseline and days 7, 15, and 30 (per-protocol set). The percentage of IgG-positive participants and 95% CI are shown. HD-TDV = high-dose formulation; TDV = approved formulation.

IgG positivity rates in baseline MNT_50_–seropositive participants were 82.4% to 85.3% at baseline, 90.1% to 90.3% at day 15, and 93.6% to 98.9% by day 30 across the vaccine groups (Figure 1). In baseline MNT_50_–seronegative participants, IgG positivity rates were 11.7% to 12.7% by day 15 across the vaccine groups, increasing to 92.7% and 60.3% by day 30 in the HD-TDV and TDV groups, respectively. Immunoglobulin M positivity rates differed by baseline MNT_50_ serostatus (Figure 2). Across both vaccine groups, the IgM positivity rates in baseline MNT_50_–seropositive participants were 6.6% and 10.5% at baseline, 6.6% and 8.4% at day 7, 24.2% and 19.4% at day 15, and 37.4% and 26.6% at day 30 in participants who received HD-TDV or TDV, respectively. In baseline MNT_50_–seronegative participants, IgM positivity rates were 0.0% and 3.8% at baseline and 0.0% and 5.1% at day 7 in participants who received HD-TDV or TDV, respectively. There was a trend for increasing rates over time, with values of 62.0% (HD-TDV) and 48.1% (TDV) at day 15, and 97.6% (HD-TDV) and 89.7% (TDV) by day 30. Similar differences were observed between the HD-TDV and TDV formulations when evaluating the IgG and IgM capture ELISA absorbance ratios (Figure 3).

**Figure 3.**
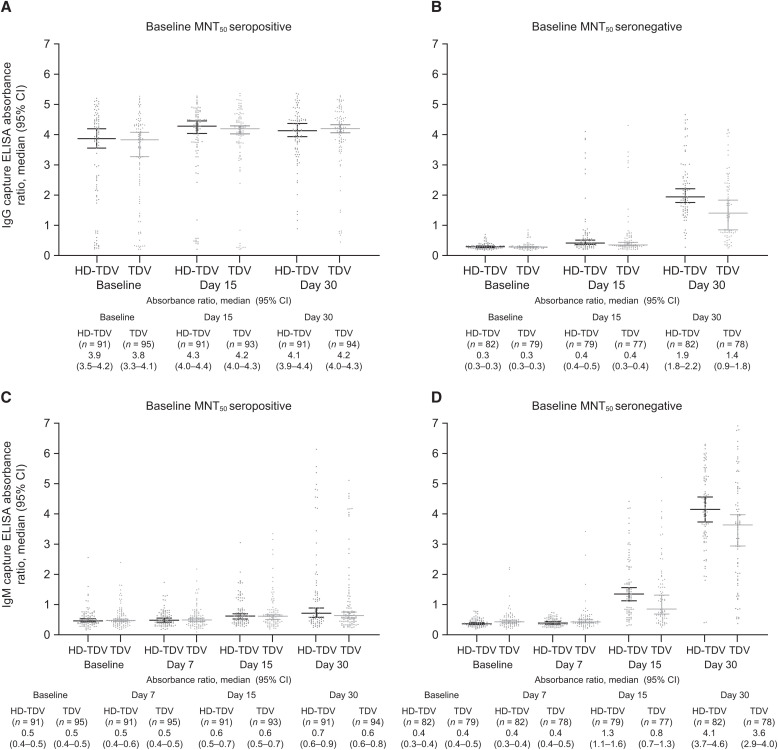
IgG (A and **B**) and IgM (**C** and **D**) absorbance ratios by baseline 50% microneutralization assay (MNT_50_) serostatus and by vaccine group at baseline and days 7 (IgM only), 15, and 30 (per-protocol set). The median IgG and IgM absorbance ratios and 95% CI are shown. HD-TDV = high-dose formulation; TDV = approved formulation.

Vaccination with TAK-003 resulted in IgG and IgM profiles consistent with neutralizing antibody geometric mean titers and seropositivity rates.^15^ These results are similar to those observed for IgG1 (which accounts for most of the IgG response) and IgM when using an indirect Luminex-based assay format at 28 days after first the dose with TAK-003.^17^

In line with IgM and IgG profiles observed in natural primary infections, IgM positivity rates in baseline MNT_50_–seronegative participants were initially low, but increased after vaccination (by day 15 in our study).^18^ Most participants were IgM positive by day 30. The presence of a few IgM-positive participants in the baseline MNT_50_–seronegative population is not unexpected, considering the differences between MNT_50_ and ELISA techniques. This might also be because of cross-reactivity to IgM antibodies produced in response to other flaviviruses. Immunoglobulin G positivity rates remained low up to day 15, but then increased rapidly by day 30. Similarly, the IgG and IgM profiles observed in natural secondary infections^18^^,^^19^ mirrored those observed in baseline MNT_50_–seropositive participants after vaccination. A high proportion of baseline MNT_50_–seropositive participants were IgG positive at baseline (82.4–85.3%), with 93.6% to 98.9% showing IgG positivity by day 30. In contrast, baseline IgM positivity rates in baseline MNT_50_–seropositive participants were low and remained lower than in those who were baseline MNT_50_ seronegative from day 15 onward. The IgG positivity rates at day 30 in baseline MNT_50_–seronegative participants receiving HD-TDV were higher compared with TDV. This difference likely reflects the 10-fold higher DENV-2 potency of HD-TDV. For the other groups and time points, the 95% CIs overlapped for HD-TDV and TDV recipients. It is of interest that the HD-TDV and TDV formulations induced a very similar initial IgM and IgG response (up to day 30), indicating that vaccine potency does not necessarily affect the qualitative IgG/IgM serological response. The absence of detectable NS1 antigen across all time points, regardless of vaccine potency, suggests that TAK-003 NS1 is either not secreted or secreted at levels below the assay limit of detection. This requires further investigation, but it should be noted that lateral flow devices are now widely used as alternative point-of-care tools for dengue diagnosis, and such assays are associated with even lower sensitivity. This study suggests that TAK-003 vaccination itself will not interfere with the interpretation of NS1 antigen–based dengue diagnostic tests. However, NS1 test sensitivity has been shown to be reduced in individuals with a natural secondary dengue infection compared with individuals with a primary infection, and this is something that may need to be considered when interpreting dengue serological tests in patients who have received a dengue vaccination.^8^^,^^9^

One limitation to our analysis is that it was performed over a short time frame; thus, antibody profiles, and their potential impact on diagnostic tests, were not assessed after the first month after vaccination. In addition, this analysis was not conducted in the context of dengue diagnostics in vaccinees presenting with febrile illness. Therefore, the impact of vaccination on test specificity and sensitivity was not established. Evidence from CYD-TDV studies^6^^,^^7^ found high rates of false positives for IgG and IgM ELISAs, particularly in the first 2 months after vaccination or in baseline MNT_50_–seronegative participants. Although specificity and sensitivity of the diagnostic tests were not assessed in our study, antibody profiles of virologically confirmed dengue cases have been evaluated in the long-term, large-scale phase 3 efficacy study,^14^ which has enrolled >20,000 children and adolescents in dengue-endemic areas of Asia and Latin America, which will allow the effect of vaccination on test performance to be assessed in this population in the future.

In summary, in the 30 days after TAK-003 vaccination, IgM and IgG profiles resembled those observed during natural dengue infections; however, no NS1 antigen was detected. These results highlight the need to consider 1) dengue vaccination status when interpreting dengue serological tests, especially in dengue-endemic countries, because IgG and IgM ELISAs may not be able to differentiate between vaccination and natural infection, and 2) the relevance of using antigen- or molecular-based assays as an alternative to identify dengue infection in febrile vaccinees.
